# Ocular Reflex Phase during Off-Vertical Axis Rotation in Humans is Modified by Head-Turn-On-Trunk Position

**DOI:** 10.1038/srep42071

**Published:** 2017-02-08

**Authors:** Samantha B. Douglas, Gilles Clément, Pierre Denise, Scott J. Wood

**Affiliations:** 1 Department of Psychology, Azusa Pacific University, Azusa CA, USA; 2 Lyon Neuroscience Research Center, CNRS UMR5292 – INSERM U1028 – University of Lyon, Impact Team, Bron, France; 3 University of Caen Normandy, INSERM COMETE, Caen, France

## Abstract

Constant velocity Off-Vertical Axis Rotation (OVAR) imposes a continuously varying orientation of the head and body relative to gravity, which generates a modulation of horizontal (conjugate and vergence), vertical, and torsional eye movements. We introduced the head-turn-on-trunk paradigm during OVAR to examine the extent to whether the modulation of these ocular reflexes is mediated by graviceptors in the head, i.e., otoliths, versus other body graviceptors. Ten human subjects were rotated in darkness about their longitudinal axis 20° off-vertical at a constant velocity of 45 and 180°/s, corresponding to 0.125 and 0.5 Hz. Binocular responses were obtained with the head and trunk aligned, and then with the head turned relative to the trunk 40° to the right or left of center. The modulation of vertical and torsional eye position was greater at 0.125 Hz while the modulation of horizontal and vergence slow phase velocity was greater at 0.5 Hz. The amplitude modulation was not significantly altered by head-on-trunk position, but the phases shifted towards alignment with the head. These results are consistent with the modulation of ocular reflexes during OVAR being primarily mediated by the otoliths in response to the sinusoidally varying linear acceleration along the interaural and naso-occipital head axis.

Constant velocity Off-Vertical Axis Rotation (OVAR) imposes a continuously varying orientation of the head and body relative to gravity. This change in orientation results in a sinusoidally varying linear acceleration that elicits both perception and ocular reflexes of tilt and translation^1^. Tilt ocular reflexes include modulation of torsional and vertical eye position that stabilize ocular alignment during roll and pitch tilt, respectively^2^. Translational ocular reflexes include modulation of horizontal and vergence slow phase eye velocity (SPV) that serve to minimize retinal slip during linear acceleration along interaural and nasooccipital axes, respectively^3^,^4^. Previous studies have demonstrated that these tilt and translation otolith-ocular reflexes during OVAR vary as a function of stimulus frequency^5^. The frequency-dependent cross over of these responses during OVAR appears to occur near the region of peak motion sickness susceptibility (~0.3 Hz)^6^.

There is evidence that the ocular reflexes during OVAR are mediated by the otoliths of the vestibular system and not the semicircular canals. Correia and Money^7^ observed that blocking the semicircular canals in cats did not eliminate the response, a finding that has been subsequently confirmed by Cohen *et al*.^8^ and Angelaki and Hess^9^ in rhesus monkeys. More importantly, Janeke *et al*.^10^ showed that partial bilateral labyrinthectomy in rabbits involving utricular nerve sections and destruction of the saccules while leaving the canals intact did eliminate the eye movement response during OVAR. Another study using rhesus monkeys showed that ocular counter-rolling (OCR) was present during both a head-on-trunk tilt and a whole head-and-trunk tilt, but no eye torsion was observed during trunk-only tilt^11^. In humans, static tilt of subjects 20° to the right or left with the head only and with the head and trunk aligned induced OCR. However, no OCR was elicited when the trunk alone was tilted^12^. Moreover, in patients with unilateral vestibular nerve sections abnormal OCR was observed when participants were tilted on the contralateral side of the lesion^13^. While the possible tilt sensitivity of the canals cannot be entirely dismissed^14^,^15^,^16^, there is strong evidence that the modulation of eye movements induced during constant velocity OVAR primarily reflects otolith function.

Nevertheless, some studies have indicated that somatosensory inputs may also play a role in the eye movements induced by constant velocity OVAR. For example, after bilateral labyrinthectomy and vestibular neurectomy in cats, neurons within the vestibular nuclei have spontaneous activity and one fourth of these neurons are still modulated by tilt^17^. Yates *et al*.^17^ suggests that this modulation demonstrates the role of somatosensory inputs, particularly from the limbs and trunk, in vestibular-mediated reflexes. Additionally, residual OCR was observed in rhesus monkeys following bilateral labyrinthectomy^11^. Whether somatosensory inputs or the otoliths primarily mediate the eye movements observed during constant velocity OVAR in humans has yet to be clarified. The purpose of the present study was to examine whether the ocular reflexes observed during constant velocity OVAR would be modified by different head-turn-on-trunk (HTOT) positions at stimulus frequencies below and above the cross over range. Because the somatosensory inputs from the trunk remain essentially the same when the head is turned to the right or the left, a shift in the phase of these ocular reflexes to remain aligned with the head in these conditions would reflect the extent to which these responses are otolith-driven.

## Methods

### Participants

A total of ten healthy human subjects (8 males, 2 females) participated in the study. The mean age of the sample was 35.40 (*SD* = 9.40), with ages ranging from 21–53. The National Aeronautics and Space Administration (NASA) institutional review board for human testing approved all test procedures. The methods were carried out in accordance with the relevant guidelines and regulations. Written informed consent was obtained from all participants prior to the commencement of testing.

### Equipment

Subjects were rotated about their longitudinal axis using an OVAR system at the NASA Johnson Space Center Neurosciences Laboratory. This rotator utilizes an electromechanical linear actuator to tilt participants along the axis of rotation from upright to 20° off vertical. Participants were secured in the chair through adjustable straps and padding at the shoulders, waist, thighs, knees and feet. Moldable firm pads (Olympic Vac-Pacs, Natus Medical Incorporated) provided constant pressure throughout the rotation while minimizing body movement relative to the chair. The head was secured to a lightweight pivoting restraint system that allowed the participant to freely turn their head toward the left or right. End stops prevented the subjects from turning more than 40° and a spring-loaded detent indicated center position. An overhead chair-fixed camera allowed the operator to monitor head position during the experiment. A chair fixed speaker minimized auditory spatial orientation cues during two-way communication between the operator and participant.

Binocular eye movements were recorded in darkness using a tightly fitting video mask (3D Video-Oculography, SensoMotoric Instruments, Inc., Boston MA) with two monochrome video cameras and near-infrared emitting diodes. The eye image field of view allowed the canthi to serve as fiducial references to verify that any movements of the camera relative to the skull during the head turns or dynamic tilt stimuli was negligible. Before testing, eye measurement calibrations were obtained by having subjects focus on wall targets at a 1.7-meter distance. These targets were positioned 5° apart over a range of ±25° horizontally and ±20° vertically. Horizontal eye targets were aligned at eye level using a laser cross-hair pointer that projected onto the calibration targets. The calibrations were used to determine the relationship between head and image coordinates, and a Cartesian rotation was applied to eye position data to correct for any misalignments of the camera system relative to the head. Following the detection of the pupil center, torsional eye movements were derived using polar cross correlation function from iris landmarks. Right-hand sign convention was used so that rightward torsion, downward, and leftward eye movements were positive. The measurements were linear over a range of ±30° in both horizontal and vertical directions with typical RMS values of less than 0.5°, and the accuracy of the torsion measures was <0.5° within the range of ±5°^18^.

Following calibration, binocular *version* (conjugate) measurements were obtained for each video field from the average of right and left eye data, while *vergence* (disjunctive) measurements were obtained from the difference between right and left eyes. Horizontal vergence was expressed in meter angles (MA), the reciprocal of fixation distance. This representation has the benefit of normalizing vergence measurements across subjects^19^. During OVAR, participants were instructed to keep their eyes open and look straight ahead.

**Figure 1:**
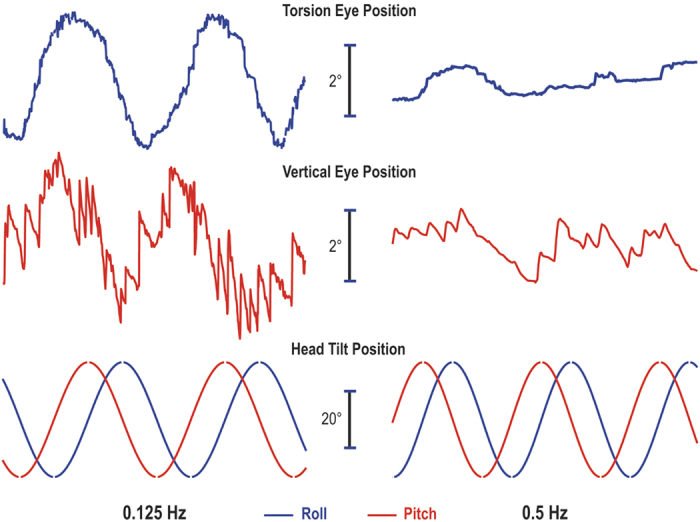
Recordings of torsional (blue) and vertical (red) eye movements in one typical subject with the head aligned with the trunk during several cycles of OVAR in darkness at both 0.125 Hz and 0.5 Hz. The torsional eye position was modulated by the continuously varying roll tilt (blue) and vertical eye position was modulated by the continuously varying pitch tilt (red).

**Figure 2:**
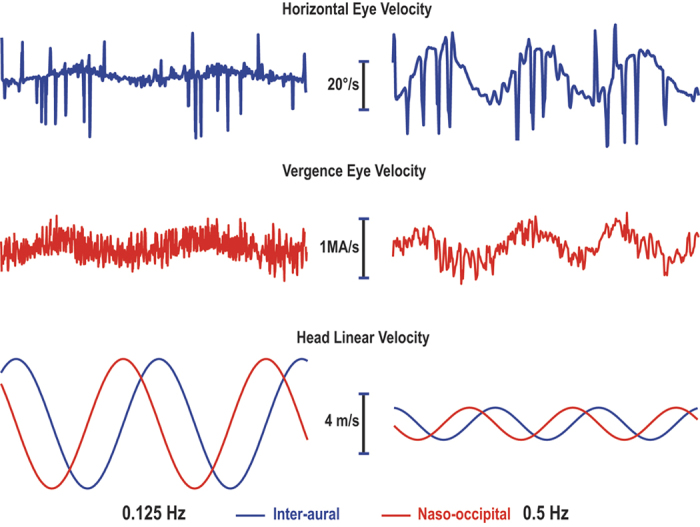
Recordings of horizontal (blue) and vergence (red) slow phase eye velocity (SPV) in one typical subject with the head aligned with the trunk during several cycles of OVAR in darkness at both 0.125 Hz and 0.5 Hz. The horizontal SPV was modulated by the sinusoidally varying linear velocity about the head inter-aural axis (blue). The vergence SPV was modulated by the sinusoidally varying linear velocity about the head naso-occipital axis (red).

Participants were rotated in darkness about their longitudinal axis 20° off vertical at 0.125 Hz (45°/s) and 0.5 Hz (180°/s). While previous studies have indicated the modulation of eye movements was consistent for both clockwise and counter-clockwise OVAR directions^6^, the direction was maintained in a counter-clockwise direction in this study. Torsion and vertical eye position phases were calculated in reference to roll and pitch tilt, respectively (Fig. 1). The equivalent peak linear acceleration at the 20° tilt position was 3.35 m/s^2^. The equivalent peak linear velocity was derived by dividing this acceleration by 2π * frequency, or 4.27 m/s at 0.125 Hz and 1.07 m/s at 0.5 Hz (Fig. 2). Horizontal and vergence slow phase velocity phases were calculated in reference to the peak linear velocity about the interaural and naso-occipital axes, respectively. Note that the peak linear velocity leads equivalent peak linear displacement (tilt position) by 90°. Eye movements were recorded after 60 seconds of constant velocity rotation to allow the per-rotary nystagmus to decay. Our experimental paradigm included measures with head aligned with the trunk and then with HTOT 40° to the right or left of center. Since the subjects rotated in a counter-clockwise direction, a leftward head turn had the effect of shifting any phases locked to the otoliths toward more leading, while a rightward head turn caused a shifting toward more lagging. Eye measures were obtained during 10 cycles at each head position and frequency, with the order of the head turns counterbalanced across subjects. After the first head turn, the head was aligned with the trunk for 10 cycles before performing a second head turn in the opposite direction.

### Data analysis

Eye position (average of left and right) was differentiated to calculate eye velocity. Representative torsional and vertical eye responses are shown in Fig. 1 for both low and high frequency conditions. Typical horizontal and vergence eye velocity are shown in Fig. 2 for both frequencies. Note that the tilt ocular responses were analyzed as a response to change in roll and pitch tilt position – also known as OCR and ocular counter-pitching. In contrast, the translational ocular responses that serve to minimize retinal slip were analyzed as a response to change in equivalent linear velocity. Specifically, the horizontal eye velocity responded to the modulation of linear velocity along the interaural axis and the vergence eye velocity responded to the modulation of linear velocity along the naso-occipital axis. The steady-state horizontal bias component normally present during low frequency OVAR^9^ would not be altered by our HTOT paradigm and was therefore excluded from our analysis.

Fast phase components of the eye movements were identified using horizontal acceleration and velocity thresholds using custom Matlab (The MathWorks Inc., Natick, MA) scripts and verified manually using horizontal, vertical and torsional eye traces to be excluded from the analysis. When present, the beginning and end points of fast phase movements were consistently aligned across all three planes of eye movement. Nonlinear least squares sinusoidal curve fits were used to describe the modulation of eye movements as a function of the sinusoidal-varying stimuli. The curve fits were used to determine the amplitude, offset, and phase of vertical and torsional eye position, and the same parameters for horizontal and vergence SPV. As described above, the phases for the tilt-ocular responses were calculated in reference to tilt position while the phases for the translational ocular responses were calculated in reference to the derived equivalent linear velocity. The difference between the eye modulation determined from the sinusoidal curve fits and the stimulus was normalized such as 0° phase was compensatory with positive leading and negative lagging^20^.

**Figure 3:**
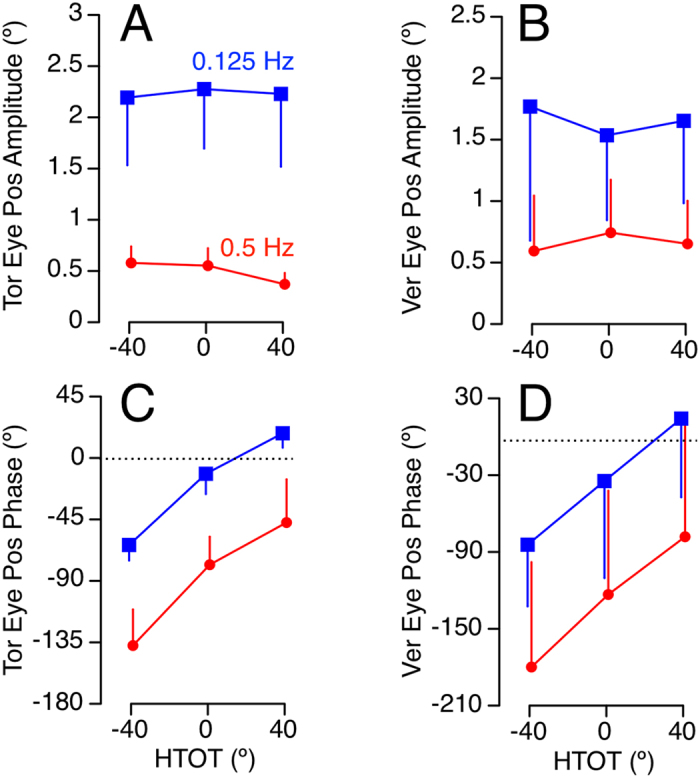
Amplitude and phase of torsional (Tor) and vertical (Ver) eye position during OVAR at 0.125 Hz and 0.5 Hz for various head-turn-on-trunk (HTOT) positions. Mean ± Standard deviation of all 10 subjects.

**Figure 4:**
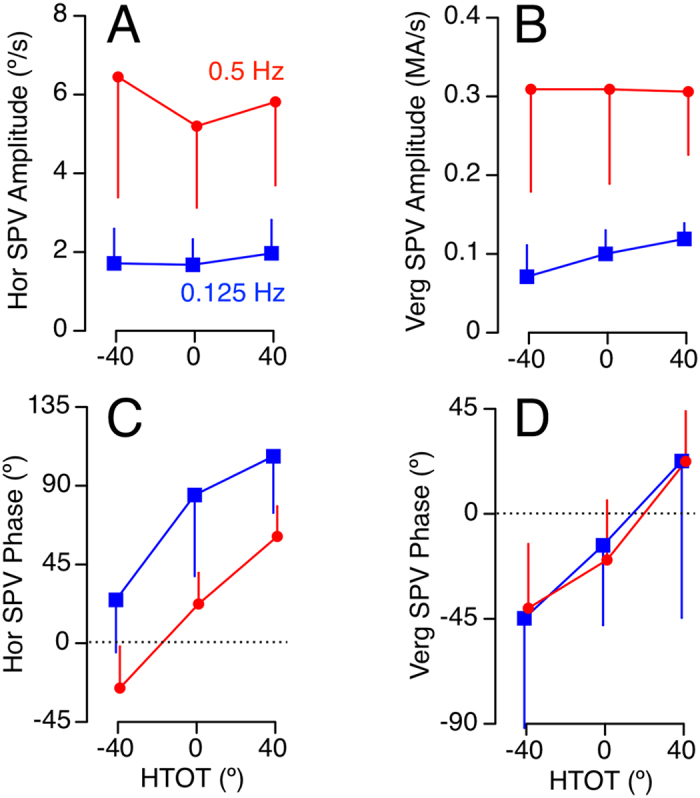
Amplitude and phase of horizontal (Hor) and vergence (Ver) slow phase eye velocity (SPV) during OVAR at 0.125 Hz and 0.5 Hz for various head-turn-on-trunk (HTOT) positions. Mean ± Standard deviation of all 10 subjects.

Shapiro-Wilk tests were used to analyze the normality of each variable. The amplitude and phases for each eye response (torsional position, vertical position, horizontal SPV, and vergence SPV) were analyzed separately using repeated measures Analysis of Variance (ANOVA) to determine whether they varied across the three HTOT positions (0°, +40°, −40°) and across both frequencies (0.125, 0.5 Hz). Post-hoc analyses to compare head aligned with both left and right HTOT positions used Bonferroni correction to reduce Type I error. When the data was not normally distributed, the Friedman test was used for the repeated measures ANOVA, and Wilcoxon Signed Rank tests with Bonferroni correction were used for post-hoc analysis. Except for Figs 3 and 4 in which standard deviations are used to reflect the inter-subject variability, all data is described using standard error of the mean to facilitate comparison of means.

## Results

The average vergence during the 0.125 Hz trials was 1.09 ± 0.01 MA and during the 0.5 Hz trials was 0.96 ± 0.01 MA. This represents a near-to-medium fixation distance sufficient to elicit translation ocular reflexes^3^. Except for vertical position, all of the amplitude measures were normally distributed. Based on the Shapiro-Wilk tests, the phase measurements that were not normally distributed included vertical position and vergence SPV at 0.125 Hz and torsion position and vergence SPV at 0.5 Hz. As described above, non-parametric tests were utilized for each of the variables identified as not normally distributed.

### Effect of frequency on amplitude

Eye responses during 0.125 Hz OVAR were dominated by a modulation of torsional and vertical eye position, compensatory for tilt relative to gravity (Fig. 1A). However, the modulation of torsion and vertical eye position was greatly reduced at 0.5 Hz (Fig. 1B). The modulation of horizontal and vergence SPV, on the other hand, was negligible at the lower stimulus frequency (Fig. 2A), but was clearly present during OVAR at 0.5 Hz (Fig. 2B). This effect of frequency on amplitude for all four eye movement responses is illustrated from the calculation of means in Figs 3A,B and 4A,B. This frequency dependent effect on response amplitude was highly significant for all four types of eye movement responses (P < 0.001).

### Effect of frequency on phase

At low stimulus frequency with the head aligned on trunk, the torsional eye position was nearly in phase with the head roll-tilt position (−11.6 ± 4.7°, Fig. 3C), while the vertical eye position was lagging head pitch tilt position (−34.6 ± 23.9°, Fig. 3D). Consistent with low-pass filtering properties, as the amplitude decreased at 0.5 Hz, the phase lag of these eye responses also increased by −66.5° for torsion position and −88.6° for vertical position (P < 0.001). The horizontal SPV phase at 0.5 Hz was 22.4° ± 5.6°. Consistent with high-pass filtering properties, as the amplitude of horizontal SPV decreased at 0.125 Hz, the phase lead also increased to 84.7° ± 14.7°, Fig. 4C, P < 0.001). This effect of frequency for torsion and vertical position and horizontal SPV was also present during the HTOT conditions. There was, however, no change in the phase of vergence SPV across the two OVAR frequencies tested (Fig. 4D). The vergence SPV phase was slightly lagging with the head aligned at both 0.125 Hz (−13.5 ± 10.8°) and 0.5 Hz (−19.78° ± 8.08°).

### Effect of HTOT on amplitude

In order to assess whether amplitude of binocular eye responses varied by HTOT, we compared changes across all three head positions. This comparison is arguably only valid at the frequencies where the responses were not negligible, i.e. at 0.125 Hz for torsion and vertical position and at 0.5 Hz for horizontal and vergence SPV. Repeated measures ANOVAs showed no significant effect of head position on vertical amplitude (Fig. 3B) at 0.125 Hz. While the repeated measures ANOVA indicated a significant effect of head position on torsional amplitude at this frequency (P = 0.045, Fig. 3A), post-hoc tests showed no significant differences between head aligned and ±40° positions. There was also no effect of HTOT on horizontal (Fig. 4A) and vergence SPV amplitude (Fig. 4B) at 0.5 Hz.

### Effect of HTOT on phase

The primary hypothesis of this study focused on whether the phase of the ocular reflexes shifted during HTOT conditions toward alignment with the head. As demonstrated in Figs 3 and 4, the phase for all four ocular responses shifted during HTOT in the expected direction and by approximately the same amplitude of the head turns, consistent with each response being modulated by the otoliths. The phase shift averaged across both HTOT positions and both frequencies was 43.0° ± 7.5° for torsion position (Fig. 3C), 50.1° ± 2.4° for vertical position (Fig. 3D), 42.2° ± 8.0° for horizontal SPV (Fig. 4C), and 32.6° ± 4.6° for vergence SPV (Fig. 4D).

Since the phase measures can be inherently unreliable when the response amplitudes are negligible, this comparison of HTOT for each ocular response was focused at the frequencies where the amplitude were more robust, namely 0.125 Hz for torsion and vertical position, and 0.5 Hz for horizontal and vergence SPV. Repeated measures ANOVA indicated that this effect of HTOT on phase was highly significant for torsion (P < 0.001), including post-hoc comparisons across each HTOT condition (Fig. 3C). The Friedman test showed that the HTOT effect of phase was also significant for vertical position at 0.125 Hz (P = 0.02, Fig. 3D). Post-hoc tests also revealed that vertical phase significantly differed between left and right head turns (P = 0.004).

For the translational ocular reflexes, the effect of HTOT on horizontal SPV phase was highly significant (P < 0.001), including post-hoc comparisons across each HTOT condition (Fig. 4C). The Friedman test indicated the HTOT effect of vergence SPV was also highly significant (P < 0.001). Post-hoc tests for vergence SPV phase showed significant differences between left and right (P = 0.002) and right and center (P = 0.002) head turns (Fig. 4D).

## Discussion

Our results clearly demonstrate that ocular reflexes are modified by HTOT during constant velocity OVAR. Specifically, the phase of each ocular reflex shifted toward alignment with the head while the amplitudes were generally not modified by HTOT. Since the somatosensory inputs from the trunk are not modified when the subjects turned their head to the left or right, the change in phase with head turn confirm that ocular reflexes are primarily mediated by the otoliths. While previous studies in lesioned animals have demonstrated the role of the otoliths during various linear acceleration stimuli^7^,^8^,^9^,^10^, our HTOT paradigm in healthy subjects generated the same conclusion in humans.

The ocular responses could have been altered by neck proprioceptive input related to the head turn itself (vestibulo-collic) or from other somatosensory receptors that are detecting the body’s orientation relative to gravity. As reviewed by Cullen^21^, proprioceptive input at the earliest stages of central vestibular processing is important for discriminating self-motion (head on trunk) versus passive motion. Beyond cervical, other somatosensory inputs related to graviception^17^ provides a means for compensation during vestibular loss or resolving potential perceptual conflicts in situations with mismatched sensory inputs^22^. Previous studies have demonstrated that somatosensory inputs can modulate the vestibulo-ocular reflex in normal healthy subjects^23^,^24^, as well as influence tilt perception^25^. Our results are consistent with the hypothesis that the ocular responses during OVAR in healthy human subjects are primarily mediated by the otoliths.

Consistent with previous studies^6^,^26^, stimulus frequency alone had a dramatic effect on each of the eye movement responses. The stimulus frequencies employed in our study (0.125 Hz and 0.5 Hz) were selected to be below and above the OVAR crossover frequency around which tilt or translational ocular reflexes dominate^6^. These reflexes serve fundamentally different functions. The tilt ocular reflexes help visual alignment between left and right eyes during static tilt or low frequency movements of the head^2^, while the translational ocular reflexes help minimize retinal slip during rapid movements^3^. Tilt ocular reflexes modulate torsion and vertical eye position as a function of linear acceleration along the interaural axis (roll tilt) and naso-occipital axis (pitch tilt), respectively. Translation ocular reflexes modulate horizontal and vergence eye velocity as a function of linear acceleration along interaural axis and naso-occipital axis, respectively.

These simultaneously occurring tilt and translational ocular reflexes reflect the inherent ambiguity in otolith afferents. It has been recognized for some time that integration of extra-vestibular sensory input is critical for resolving this ambiguity^27^,^28^. In addition to this multisensory integration solution, Mayne proposed that a frequency segregation of otolith inputs might serve to provide for a rapid response when needed^27^. Paige^19^ suggested that attributing low frequency input to tilt and high frequency input to translations appears consistent with natural behavior. The effects of stimulus frequency on multidimensional VOR responses to linear acceleration in the absence of concomitant canal or visual inputs during OVAR is consistent with observations during translational sled motion^29^ and variable radius centrifugation^26^. In the case of OVAR, the equivalent linear acceleration varying in both roll and pitch planes during the change in orientation occurs without the canal inputs that are present during natural roll and pitch tilts.

While a phase difference across 0.125 Hz and 0.5 Hz was present for horizontal SPV (Fig. 4C), there was no apparent change in phase of vergence SPV between these two frequencies (Fig. 4D). There is a known interaction between vertical eye position and vergence, with a consistent tendency for the eyes to deviate downward during near vergence^30^. At low frequency, the modulation of vertical eye position may have induced a modulation of vergence based on this interaction. With the head aligned on trunk, the phase lag of the vertical eye position may have influenced the modulation of vergence position, and therefore the vergence SPV. At high frequency, the modulation of vertical eye position is negligible and would therefore be less of a factor.

For both tilt and translation ocular responses, the HTOT paradigm demonstrated that phase was a function of head position relative to gravitational vertical rather than body position relative to vertical. During constant velocity OVAR, a head turn to the right or left directly influenced the phase of each response. Since the peak-to-peak linear acceleration is the same during the head turn, it is not surprising that the amplitude of the ocular responses was not altered. Although the role of other sensory inputs cannot be dismissed^24^, the eye movements generated by the sinusoidally varying linear acceleration during constant velocity OVAR are primarily mediated by the graviceptors in the head, i.e., otoliths, versus other body graviceptors^25^.

The HTOT paradigm in healthy subjects proved to be useful for examining the otolith contribution to ocular reflexes during OVAR. This same paradigm has potential to examine the otolith contribution to motion perception, which is known to have different phase characteristics than eye movements^20^,^31^. In addition, cardio and respiratory autonomic reflexes could be explored with HTOT paradigm to separate out otolith contributions from other sensory inputs^32^,^33^.

Several models have accurately predicted tilt and translational ocular reflexes based on visual and vestibular contributions to the central processing^26^,^34^. The assumption that other sensory inputs are minimally contributing to ocular reflexes in healthy subjects is consistent with our results. One limitation of this study is that our results were obtained in healthy normal subjects, and therefore may not be generalizable to patients suffering from sensory pathology. Following vestibular loss, sensory substitution is known to occur that may increase the contributions of somatosensory input^17^. Repeating the HTOT paradigm during OVAR in patients with chronic bilateral vestibular loss will help determine the extent to which additional sensory inputs need to be factored in models that account for sensory substitution processes for otolith ocular reflexes.

The diagnostic sensitivity and specificity of OVAR to detect otolith dysfunction could be enhanced by the HTOT paradigm. In addition, it is important to consider that otolith reflexes are not robust at all frequencies^6^. During OVAR at low frequencies it would be preferable to use the modulation of torsion and/or vertical eye position as a measure of otolith function. In contrast, during OVAR at higher frequencies horizontal and/or vergence SPV would be the best measure of otolith function. By considering this relationship between the modulation of otolith ocular reflexes and stimulus frequency, with the addition of the HTOT paradigm, researchers and clinicians can improve the reliability of using OVAR as a test of otolith function.

## Additional Information

**How to cite this article**: Douglas, S. B. *et al*. Ocular Reflex Phase during Off-Vertical Axis Rotation in Humans is Modified by Head-Turn-On-Trunk Position. *Sci. Rep.* **7**, 42071; doi: 10.1038/srep42071 (2017).

**Publisher's note:** Springer Nature remains neutral with regard to jurisdictional claims in published maps and institutional affiliations.
